# Supplementary material for: Novel Ratio Soluble Fms-like Tyrosine Kinase-1/Angiotensin-II (sFlt-1/ANG-II) in Pregnant Women Is Associated with Critical Illness in COVID-19
Source: Viruses. 2021 Sep 23;13(10):1906. doi: 10.3390/v13101906 (PMC8538263; doi:10.3390/v13101906)
Supplement: Supplementary file 1 [file viruses-13-01906-s001.zip › viruses-1370774-supplementary.pdf]

Supplementary Materials

Table S1. Pregnancy outcome of the study population.

Outcome	Non-severe COVID-19 n= 55	Severe COVID-19 n= 25	p-value
Preeclampsia	6 (10.9%)	2 (8.0%)	0.118
Delivery			
Cesarean section	18 (32.72%)	24 (96%)	<0.0001
Vaginal	37 (67.27%)	1 (4%)	
Birth weight (grams)	2890 (2350-3200)	2612 (1647-2985)	0.114
Apgar score 1 min	8 (7-8)	7 (5-8)	0.046
Apgar score 5 min	9 (8-9)	8 (7-9)	0.027
Neonatal asphyxia	1 (1.81%)	6 (24%)	<0.0001
RDS	4 (7.27%)	10 (40%)	0.001
Neonatal sepsis	12 (21.81%)	7 (28%)	0.617
NICU admission	2 (3.63%)	11 (44%)	<0.0001
Neonatal death	1 (1.81%)	7 (28%)	<0.0001

RDS: respiratory distress syndrome; NICU: neonatal intensive care unit; Neonatal asphyxia was defined as an umbilical arterial pH <7.1 or 5-minutes Apgar score <6.

Table S2. Association between sFlt-1/ANG-II ratio and each adverse outcome of COVID-19.

Outcome	sFlt-1/ANG-II ratio		
	OR	95%CI	p-value
Severe pneumonia	1.31	1.09-1.56	0.003
ICU admission	1.05	1.01-1.09	0.007
Intubation	1.09	1.02-1.16	0.008
Viral sepsis	1.04	1.01-1.08	0.008
Maternal death	1.04	1.00-1.07	0.018

Soluble fms-like tyrosine kinase-1; ANG-II: Angiotensin-II; ICU: Intensive care unit; OR: Odds ratio; CI: Confidence interval.
